# Enlightenment of Yeast Mitochondrial Homoplasmy: Diversified Roles of Gene Conversion

**DOI:** 10.3390/genes2010169

**Published:** 2011-02-12

**Authors:** Feng Ling, Tsutomu Mikawa, Takehiko Shibata

**Affiliations:** 1 Chemical Genetics Laboratory, RIKEN Advanced Science Institute/2-1 Hirosawa, Wako-shi, Saitama 351-0198, Japan; E-Mail: ling@postman.riken.go.jp; 2 Biometal Science Laboratory, RIKEN SPring-8 Center/Mikazuki cho, Hyogo 679-5148 Japan; E-Mail: mikawa@riken.jp; 3 Division of Molecular and Cellular Physiology, Department of Supramolecular Biology, Graduate School of Nanobiosciences, Yokohama City University/1-7-29 Suehiro cho, Tsurumi-ku, Yokohama, Kanagawa 230-0045, Japan; 4 Cellular & Molecular Biology Laboratory, RIKEN Advanced Science Institute/2-1 Hirosawa, Wako-shi, Saitama 351-0198, Japan

**Keywords:** rolling circle replication, homologous pairing, D-loops, RecA/Rad51 family proteins, Rad52, Mhr1, Ntg1, endonuclease III homologues, concerted evolution, sequence homogenization

## Abstract

Mitochondria have their own genomic DNA. Unlike the nuclear genome, each cell contains hundreds to thousands of copies of mitochondrial DNA (mtDNA). The copies of mtDNA tend to have heterogeneous sequences, due to the high frequency of mutagenesis, but are quickly homogenized within a cell (“homoplasmy”) during vegetative cell growth or through a few sexual generations. Heteroplasmy is strongly associated with mitochondrial diseases, diabetes and aging. Recent studies revealed that the yeast cell has the machinery to homogenize mtDNA, using a common DNA processing pathway with gene conversion; *i.e.*, both genetic events are initiated by a double-stranded break, which is processed into 3′ single-stranded tails. One of the tails is base-paired with the complementary sequence of the recipient double-stranded DNA to form a D-loop (homologous pairing), in which repair DNA synthesis is initiated to restore the sequence lost by the breakage. Gene conversion generates sequence diversity, depending on the divergence between the donor and recipient sequences, especially when it occurs among a number of copies of a DNA sequence family with some sequence variations, such as in immunoglobulin diversification in chicken. MtDNA can be regarded as a sequence family, in which the members tend to be diversified by a high frequency of spontaneous mutagenesis. Thus, it would be interesting to determine why and how double-stranded breakage and D-loop formation induce sequence homogenization in mitochondria and sequence diversification in nuclear DNA. We will review the mechanisms and roles of mtDNA homoplasmy, in contrast to nuclear gene conversion, which diversifies gene and genome sequences, to provide clues toward understanding how the common DNA processing pathway results in such divergent outcomes.

## 1. Introduction

In human postmitotic cells, such as muscle and nerve cells, the mtDNA accumulates various mutations during the aging process and becomes heteroplasmic, and the resultant mitochondrial dysfunctions are considered to contribute to age-related degenerative diseases [1,2]. However, heteroplasmy, even that consisting of silent mutations, segregates within a few generations into homoplasmy through a putative genetic bottleneck, for which the genetic or protein-elements have not been identified [3–7]. Maternally inherited heteroplasmic mtDNA mutations are considered to cause some mitochondrial diseases [8] and diabetes [9]. Thus, homoplasmy is very important to health. Heteroplasmic yeast zygotes are readily obtained in the laboratory by mating **a** cells and *α* cells containing different mitochondrial genetic markers. The heteroplasmic yeast cells segregate the homoplasmic progeny during zygote outgrowth in the absence of any selective pressure, and after ten to twenty generations, all of the cells in the culture are homoplasmic [10]. A mtDNA gene conversion deficient mutant (*mhr1-1*) was isolated [11] and characterized, and revealed that Mhr1 is essential for mtDNA partitioning into daughter cells [12], and functions in the homogenization of heteroplasmic mtDNA to generate homoplasmic progeny [13]. The study further showed that Mhr1, like RecA, has an activity to form D-loops from homologous single-stranded DNA and double-stranded DNA, but unlike RecA, it does not require ATP [12]. The *mhr1-1* mutation causes a single amino acid replacement that inactivates the D-loop forming activity of Mhr1, and thus Mhr1 plays a role in mtDNA partitioning and homoplasmy, as well as mtDNA gene conversion.

Gene conversion is a type of homologous (DNA) recombination, in which the sequence of the recipient DNA is replaced by copying the homologous sequence of the donor DNA. The other type of homologous recombination is crossing-over, in which parts of a pair of homologous chromosomes are reciprocally exchanged at mutually homologous sequences. The primary role of gene conversion is to repair DNA double-stranded breaks, in which the sequence flanking the damage is replaced by a copy of the homologous DNA sequence. Gene conversion is occasionally associated with crossing-over.

**Figure 1:**
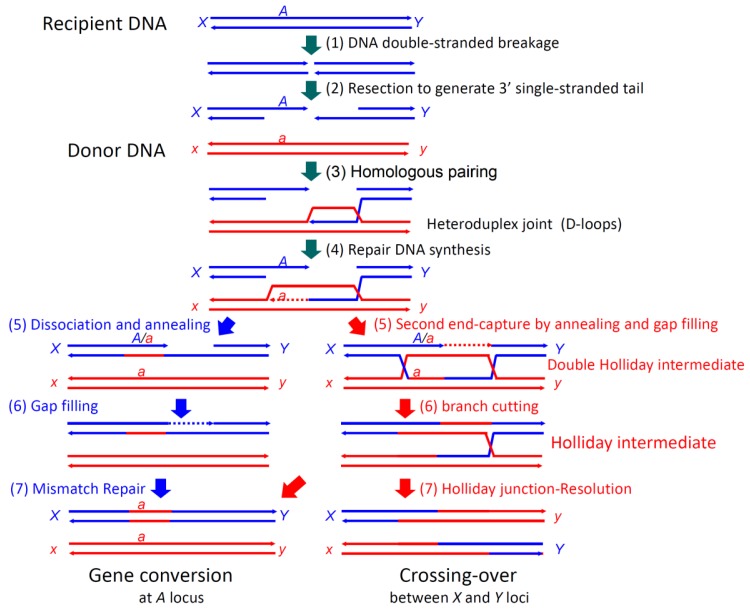
Pathways of homologous recombination. Both sides of a double-stranded break (Step 1) are resected to generate 3′ single-stranded tails at Step 2. At Step 3, the single-stranded tail derived from the first end finds a complementary sequence within the homologous DNA and forms a heteroduplex with a D-loop (homologous pairing), followed by repair synthesis to restore the broken sequence from the paired 3′ single-stranded tail at Step 4. DNA synthesis replaces the parental DNA strand to enlarge the D-loop. After Step 5, the double-stranded break-repair pathway is illustrated on the right and the synthesis-dependent strand-annealing (SDSA) pathway is on the left. In the double-stranded break-repair pathway, the second end is captured by annealing with the D-loop at Step 5. The following gap filling and branch migration generate a double-Holliday intermediate. Cleavage of one of the inter-crossed strands leads to the Holliday intermediate at Step 6. At Step 7, the Holliday intermediate can be resolved in two ways, by cutting either the outer strands, to generate the crossing-over product, or the inter-crossed strands, to generate the gene conversion product after mismatch repair. In the SDSA pathway, the synthesized strand is dissociated from the double-stranded DNA and anneals with the second end at Step 5, followed by gap filling (Step 6) and mismatch repair (Step 7) to generate only a gene conversion product. *A* and *a*, *X* and *x*, and *Y* and *y* are alleles at the *A*, *X* and *Y* loci, respectively.

Various DNA lesions and erroneously incorporated bases in double-stranded DNA are repaired by the use of the complementary strand as the template, in nucleotide-or base-excision repair. However, excision repair cannot repair DNA double-stranded breaks and single-stranded gaps that contain damaged bases, since no intramolecular template for repair is available. Thus, these DNA lesions are repaired by homologous recombination using an identical or homologous DNA sequence, such as a sister or homologous chromosome, as the template for repair, or by simple end-joining, in a reaction called non-homologous end-joining (NHEJ). NHEJ is associated with mutations, while the repair of double-stranded DNA-breaks by homologous recombination is much more accurate.

In double-strand break-repair by homologous recombination (Figure 1) [14], both ends of a double-stranded break are resected to generate 3′ single-stranded tails. A RecA-family protein (RecA in eubacteria and Rad51 or Dmc1 in eukaryotes) pairs one of the tails (of the first end) with the complementary sequence of a donor double-stranded DNA (homologous pairing), in an ATP-dependent manner, to form a D-loop (displacement loop), in which the parental strand is displaced by the invading, single-stranded tail [15–19]. Once the single-stranded tail is formed in the cells, it is covered by single-strand binding protein, SSB in eubacteria and RPA (replication protein A) in eukaryotes, which is required for efficient homologous pairing, but inhibits the initial binding of RecA-family proteins to the single-stranded tail. Rad52 was first identified as a recombination mediator, which loads a RecA-family protein onto the SSB-or RPA-coated single-stranded tails [20].

Repair DNA synthesis then starts at the 3′ terminus of the single-stranded tail in the D-loop, to copy the complementary sequence of the donor DNA and recover the sequence lost by the double-stranded breakage. The following second end capture and gap filling with branch migration generate a double Holliday intermediate [21], which is processed into a Holliday intermediate. This intermediate is further processed by mismatch repair and junction-resolution into gene conversion products and/or crossing-over products, as R. Holliday postulated [22]. The synthesis-dependent single-strand annealing (SDSA) mechanism is another pathway for gene conversion without associated crossing-over, in which the extended single-stranded tail of the first end is released from the template and anneals with the single-stranded tail of the second end, followed by gap filling and removal of excess sequences (Figure 1) [23].

In this review, we will discuss the mechanisms and roles of mtDNA homoplasmy, in contrast to nuclear gene conversion, which diversifies gene and genome sequences, to clarify how the common DNA processing pathway plays various genetic roles and results in such divergent outcomes.

## 2. Gene Conversion in Nuclear Genome

### 2.1. Gene Conversion Generates Diversity in a DNA Sequence Family in the Nuclear Genome

The role of meiotic homologous recombination, either crossing-over or gene conversion, is the acquisition of genetic diversity in each species by reorganizing parental alleles, to facilitate adaptation to environmental changes. In meiosis, the general process to produce haploid cells from diploid mother cells, chromosome duplication is followed by two rounds of cell division without DNA replication. Gene conversion and crossing-over are induced at very high frequencies in the early phase of the first meiotic cell division, by site-and timing-specific double-stranded breakage catalyzed by SPO11. Crossing-over is essential for the precise sorting of each of the homologous chromosome pairs to the opposite poles in the first meiotic cell division, to accomplish the meiotic segregation of homologous chromosomes. This is supported by the observations that most meiotic recombination-deficient mutants cause meiotic nondisjunction, to produce aneuploids. In human, reduced meiotic recombination induces nondisjunction of chromosome 21, which causes Down Syndrome [24].

Meiotic crossing-over is strictly restricted between alleles, and generates various new combinations of alleles derived from both parents to increase genetic diversity. Crossing-over between nonallelic genes necessarily results in chromosomal aberrations, but gene conversion occurs between not only alleles but also non-allelic genes with similar sequences (*homologous* recombination), and consequently, occasionally produces chimeric genes with additional genetic variations. Both meiotic crossing-over and gene conversion depend on RecA-family proteins, Rad51 and Dmc1, as well as a recombination mediator (Rad52 in *S. cerevisiae*).

Somatic gene conversion among the members of a DNA sequence family with some sequence variations results in extensive sequence diversification. An example is the production of genes encoding immunoglobulins with various antigen-specificities in a chicken cell line (DT40), derived from bursa B cells [25–27]. This gene conversion allows a 4–20% level of mismatched base-pairs between the donor and recipient DNA sequences [28], which is orders of magnitude higher than the levels tolerated by meiotic gene conversion and double-stranded break-repair observed in somatic cells. The hypervariable region is also a hotspot of somatic hypermutation, and may contribute to immunoglobulin diversification. When DT40 cells were treated with trichostatin A, a histone deacetylase inhibitor, almost all of the cells within the culture recombined at the loci under the optimal conditions, and the induced recombination was suppressed simply by removing the drug. This finding enabled the development of a quick method to generate clones of functional antibodies (ADLib system) [29]. DNA sequence analyses of these established cell lines revealed that the genes encoding the newly obtained, functional antibodies were mostly generated by gene conversion, and the contribution of somatic hypermutation was very limited, even though the trichostatin A-treatment also enhanced somatic hypermutation [29]. Details about the rapid generation of specific antibodies by the ADLib system are described in the review by Kunihiro Ohta, in this issue. The results obtained with the ADLib system suggest that gene conversion between similar sequences of a DNA sequence family has significant power to diversify genes and even to create genes encoding proteins with new functions, as discussed previously for DNA shuffling [30].

Each cell contains a number of mtDNA copies, which suffer from a high frequency of mutagenesis and are recombined to generate either gene conversion products or crossing-over products in yeast and other organisms [10]. Thus, it might be expected that the sequence of mtDNA would be extensively diversified, but as mentioned, mtDNA maintains homoplasmy. This suggests that mitochondria have potent mechanisms to suppress the diversification of mtDNA and to maintain mtDNA homoplasmy.

### 2.2. Double-Stranded Breaks and Homologous Pairing also Induce DNA Replication

**Figure 2:**
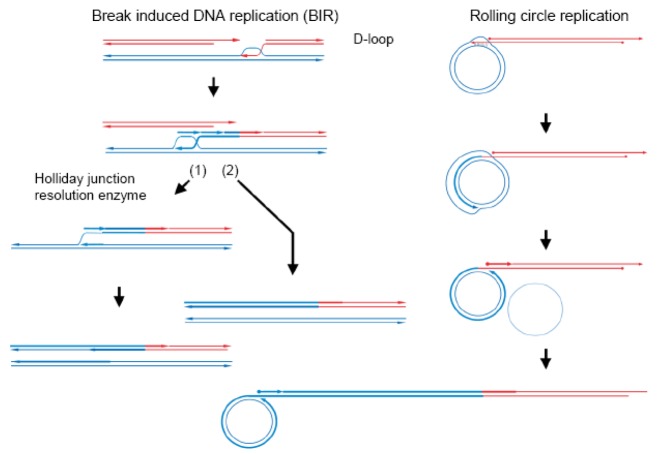
Double-stranded break-induced DNA replication (BIR) and rolling circle DNA replication. Steps after homologous pairing (D-loop formation; see Figure 1) are indicated. In pathway (1), the crossed strands are cleaved and a replication fork is formed, and then authentic DNA replication occurs. In pathway (2), the D-loop moves along with strand synthesis, followed by lagging strand synthesis. When the donor DNA is circular, the D-loop formation initiates rolling circle replication. Bold lines indicate newly synthesized strands. Red DNA alleles are either lost by double-stranded break-induced DNA replication, or extensively decreased within a multicopy DNA population by rolling circle replication.

Occasionally, repair synthesis that starts at a D-loop continues without capturing the second end, with associated lagging strand DNA synthesis, as first shown in bacteriophage T4 [31], bacteria [32,33] and yeast [34]. This prolonged DNA synthesis (double-stranded break induced DNA-replication, often abbreviated as BIR) in yeast was shown to cause coconversion to the donor genotype (Figure 2) [35,36]. Double-stranded break-induced DNA replication occurs when the second end is not available, as in the case of a collapsed replication fork, which is recovered by this double-stranded break-induced DNA replication-dependent mechanism [37]. When the second end was available in *S. cerevisiae*, most of the double-stranded breaks induced in diploid wild-type cells were repaired by Rad51-dependent gene conversion, but when the double-stranded breaks were induced in diploid *rad51*Δ*/ad51*Δ cells, one-third of the breaks were repaired by double-stranded break-induced DNA replication, while none was repaired by simple gene conversion. This double-stranded break-induced DNA replication mechanism absolutely depends on Rad52, but does not require Rad51 [34].

## 3. Yeast *Saccharomyces cerevisiae* Mitochondria Have a Rolling Circle DNA Replication-Dependent Mechanism to Recover and Maintain mtDNA Homoplasmy

### 3.1. Gene Conversion in Yeast Mitochondria

MtDNA recombination, involving crossing-over and gene conversion, has been intensively studied and well characterized in the yeast *Saccharomyces cerevisiae* [10]. Some sequence-specific endonucleases, such as Endo.SceI [38,39] and I-SceI [40,41], were shown to induce very efficient gene conversion at their cleavage sites in mtDNA, and the cleaved DNA always acted as the recipient, as also observed in nuclear gene conversion. Endo.SceI is a heterodimer of Ens2, encoded by a mitochondrial gene, and a nuclear chromosome-encoded mitochondrial 70 kDa heat shock protein (mtHSP70) [42], and numerous cleavage sites for Endo.SceI exist in the yeast mtDNA [39]. On the other hand, I-SceI and other mitochondrial sequence-specific endonucleases (homing endonucleases) are homodimers encoded within mitochondrial introns, functioning in the transposition of the introns into their intronless alleles (homing) through gene conversion, and each has a rare cleavage site within its intronless allele [40]. The sequence-specific endonuclease-induced gene conversion induced by I-SceI is the most efficient; *i.e.*, all progeny of the mating of I-SceI intron-plus cells and I-SceI intronless cells are I-SceI intron-plus. Note that I-SceI is the name of an intron of the 21S ribosomal RNA gene as well as the name of the sequence-specific endonuclease encoded in the intron [43], and that I-SceI and Endo.SceI [38] are different endonucleases with diverse target specificities.

Until the mid-nineties, the elucidation of the molecular mechanisms of homologous mtDNA recombination had been hampered by unsuccessful attempts to isolate mtDNA recombination-deficient mutants, until the identification of the first defective yeast mutant, *mhr1-1*. By using the efficient mtDNA gene conversion induced by I-SceI or Endo.SceI and developing a “mitochondrial crossing in haploid” screening system, a homologous mtDNA recombination-defective mutant was isolated for the first time [11]. The mutation is a recessive, single base-substitution in a nuclear gene, *MHR1*, and it generates an amino acid-substitution in the mitochondrial protein, Mhr1 [44].

The *mhr1-1* mutation largely suppresses the gene conversion induced by I-SceI and Endo.SceI [11]. Although only a mild reduction in mtDNA crossing-over was observed in *mhr1-1* cells [11], this is due to the crossing-over between unlinked mtDNA markers, and subsequent physical tests revealed that *mhr1-1* clearly decreased mtDNA crossing-over [45].

Before *mhr1-1* was found, two genes or proteins that may be related to mtDNA recombination were known. Pif1 is a DNA helicase [46], and its mutant was isolated by its effect on recombination between a specified type of ρ^−^ DNA (petite; respiration defective mtDNA with a large deletion) and ρ^+^ (respiration proficient) mtDNA, but this mutation does not affect recombination between ρ^+^ mtDNAs [47,48]. Pif1 also functions in nuclei at telomere [49] and minisatellite DNA [50]. Cce1 encodes a DNA endonuclease that resolves the Holliday junction *in vitro*, but a recombination-deficient phenotype was not reported [51,52].

### 3.2. Rolling Circle DNA Replication as a Mechanism for mtDNA Inheritance and Homoplasmy in Yeast

*MHR1* encodes a protein that catalyzes D-loop formation from homologous double-stranded DNA and linear single-stranded DNA in the absence of ATP. The *mhr1-1* mutation is a single amino acid-substitution causing the inactivation of the D-loop forming activity of Mhr1 [12]. Thus, the D-loop forming activity of Mhr1 explains the deficiencies of *mhr1-1* in homologous mtDNA recombination, either gene conversion or crossing-over. The *mhr1-1* mutation generates a pleiotropic phenotype in ρ^+^ cells. First, it showed defective mtDNA maintenance at a higher temperature, with associated defects in replicated mtDNA transmission into daughter cells (temperature sensitive partitioning) [11,12]. Second, *mhr1-1* exhibited slower segregation of heteroplasmic cells to generate homoplasmic cells at a sub-lethal temperature, and the overexpression of Mhr1 in ρ^+^ cells accelerated the segregation of heteroplasmic cells, indicating that Mhr1 plays an important role in mtDNA homogenization to maintain homoplasmy [13].

**Figure 3:**
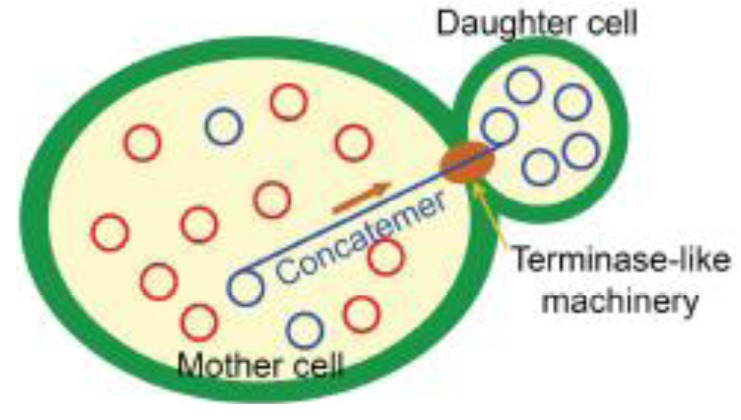
Mechanism for the efficient segregation of heteroplasmic cells in the yeast, *S. cerevisiae*. In the heteroplasmic mother cell, a randomly selected template circular DNA molecule (a blue circle in “Mother cell”) is replicated in a rolling circle mode to produce concatemers. The concatemers are selectively transmitted to daughter cells by a mechanism resembling that of terminases, which are ATP-dependent motor proteins that package a single phage genome unit from the phage DNA concatemers into the phage capsid [54]. Upon transmission into the daughter cells, the concatemers are processed into circular monomers (blue circles in “Daughter cell”), as in the case of phage DNA packaging [12] and constitute homoplasmic mtDNA.

The mechanistic roles of Mhr1 in mtDNA partitioning into daughter cells were revealed when the ρ^+^ mtDNA (*ca.* 80 kbp) was analyzed in mother cells and growing buds separated from dividing cells, by the use of pulsed-field gel electrophoresis. The major species of mtDNA in the mother cells was multimers, as observed in whole cells, but in the bud, the major mtDNA species was monomers [12]. Detailed information about the forms of mtDNA in the mother and daughter cells was obtained by the analysis of small ρ^−^ mtDNA (*ca.* 10 kbp) by 1D and 2D agarose gel-electrophoresis. The major species of mtDNA in cells are linear head-to-tail multimers of varying sizes (concatemers) in the mother cells, and circular monomers in daughter cells [12,13]. The amounts of ρ^−^ mtDNA concatemers depend on the activity of Mhr1 within cells, as revealed by the effects of *mhr1-1* and the overexpression of Mhr1 in yeast cells [13]. These features are very similar to those observed in the late phase of DNA replication and packaging of *E. coli* phages lambda and *E. coli* phage T4 DNA replication; *i.e.*, recombination-dependent formation of concatemers, and selective packing of concatemers into phage capsids by terminase [31,53]. The defective partitioning of mtDNA in *mhr1-1* yeast cells indicates that the concatemers formed by Mhr1-dependent mtDNA replication are an essential intermediate for partitioning promoted by the terminase-like enzyme (Figure 3).

Concatemers are formed from circular monomers of DNA, by either crossing-over type homologous recombination or rolling circle DNA replication. A series of pulse labeling and chase experiments of ρ^−^ mtDNA in synchronized cells supported rolling circle DNA replication and excluded crossing-over as the major pathway of concatemer formation in the mother cells, and also showed that the concatemers in the mother cells are the precursors of the circular mtDNA monomers in the growing buds [13]. These results indicated that rolling circle DNA replication is the major pathway to form ρ^−^ mtDNA concatemers, which are selectively transmitted into buds [12,13]. Thus, yeast cells have the Mhr1-dependent mechanism to replicate mtDNA in the rolling circle mode, to form concatemers and to transmit them selectively into daughter cells (mtDNA partitioning). These Mhr1-dependent mechanisms provide an explanation for the homogenization of heteroplasmic ρ^+^ mtDNA to generate homoplasmic ρ^+^ cells (Figure 3): Mhr1-dependent rolling circle replication allows the progeny of a template mtDNA molecule to dominate in a multicopy mtDNA population within a cell, and consequently, heteroplasmic mtDNA is rapidly homogenized during mitotic yeast cell growth to generate homoplasmic cells. In addition, the selective transmission of concatemers, the products of rolling circle replication, to daughter cells further enhances mtDNA homogenization [13].

### 3.3. Initiation of Rolling Circle mtDNA Replication by Site-Specific Double-Stranded Breakage, Catalyzed by a Base-Excision Enzyme for the Repair of Oxidatively Damaged DNA

In *S cerevisiae* ρ^−^ mitochondria, rolling circle DNA replication is initiated by a double-stranded break introduced at a mtDNA replication origin, such as *ori5* [45]. The composition of the yeast mtDNA replication origin resembles that of the human heavy chain replication origin [55,56]; *i.e.*, the sequence overlaps a transcription promoter and is very AT-rich, with three GC-rich clusters. The yeast hypersuppressive ρ^−^ mtDNA has been used as an excellent model system for studying the mechanisms of mtDNA replication and its initiation. Hypersuppressive ρ^−^ mtDNA is a small fragment (1 kbp or less) of ρ^+^ mtDNA (*ca.* 80 kbp) that contains an active mtDNA replication origin, and it has an extreme selective advantage over ρ^+^ mtDNA or neutral ρ^−^ mtDNA. Among the yeast mtDNA replication origins, *ori5* is the most active [55,57]. The selective replication and propagation of the hypersuppressive ρ^−^ mtDNA over ρ^+^ mtDNA or neutral ρ^−^ mtDNA depends on Mhr1 [45], but does not require the transcriptional RNA polymerase (Rpo41) of mitochondria [58]. These observations provide additional support for Mhr1-initiated rolling circle replication, but not for RNA-primed replication, as the major mechanism for yeast mtDNA replication. Rolling circle replication should be associated with lagging strand synthesis, and a primase has not been found in any mitochondria. This observation also suggests that Rpo41 does not act as a primase for lagging strand synthesis.

A series of biochemical and genetic analyses of a hypersuppressive ρ^−^ mtDNA containing *ori5* revealed that Ntg1 by itself introduced a double-stranded break in *ori5*, in mtDNA isolated from oxidatively stressed yeast cells *in vitro*. In addition, the double-stranded break introduced by Ntg1 *in vivo* is responsible for the initiation of rolling circle DNA replication, the selective replication and propagation of hypersuppressive ρ^−^ mtDNA, and crossing-over in yeast mitochondria [45]. These findings are somewhat surprising, since Ntg1, a homologue of *E. coli* endonuclease III, is a well-characterized base-excision repair enzyme that introduces a single-stranded break at nucleotides with an oxidatively damaged base, by its DNA-N-glycosylase- and AP-site-specific DNA-lyase activities [59]. The ability of Ntg1 to cause double-stranded breaks was a novel finding. The Ntg1 treatment of plasmid DNA isolated from heavily oxidatively stressed *E. coli* cells, under the same conditions, used for the detection of a double-stranded break at *ori5*, generated only single-stranded breaks, but no double-stranded breaks [45]. These results suggest that mtDNA has a specific structure at the mtDNA replication origin, *ori5*, for the regulation of mtDNA replication initiation under oxidatively stressful conditions.

### 3.4. Regulation of the Initiation of Rolling Circle Replication and Somatic Homologous Recombination of mtDNA by ROS in Yeast

MtDNA replication is not coupled with the cell division cycle. The mtDNA copy number changes in response to physiological conditions. Oxidative stress is known to increase the mtDNA copy number in mammals. However, the mechanisms regulating mtDNA replication and copy number are not known [60,61]. The requirement for Ntg1 in the initiation of rolling circle DNA replication suggests that reactive oxygen species (ROS) act directly as signaling mediators to regulate the yeast mtDNA replication. This assumption was confirmed by studies using isolated mitochondria and intact cells with the hypersuppressive ρ^−^ mtDNA. When an appropriate type of oxidative stress was applied to isolated mitochondria, the levels of the *ori5*-specific double-stranded break and the copy number of the hypersuppressive ρ^−^ mtDNA simultaneously increased [45,62]. Since isolated mitochondria were used in these studies, the effects of nuclear gene expression and metabolic systems could be ignored. Ntg1 is able to recognize an oxidized base on single-stranded DNA and to cleave the DNA at the site, and the mtDNA replication origin (*ori5*) has a local single-stranded structure *in vivo*, as shown by the locus-specific sensitivity to the single-stranded DNA- specific S1 nuclease and resistance to double-stranded DNA-specific restriction endonucleases [62].

**Figure 4:**
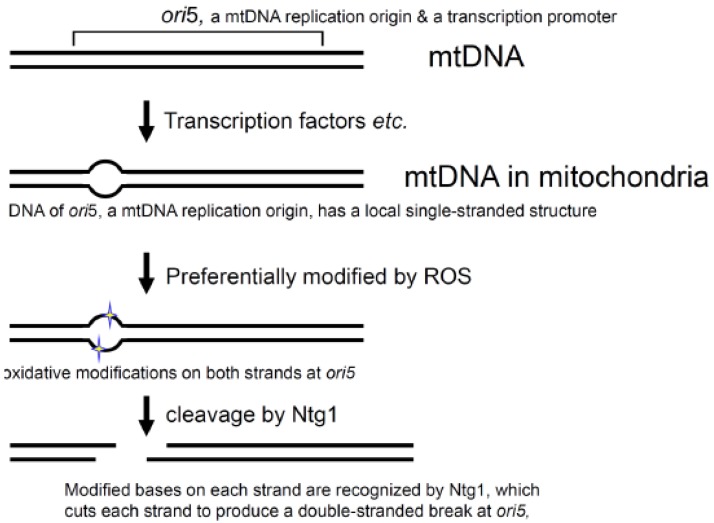
ROS mediated double-stranded breakage at the mtDNA replication origin, *ori5*.

Since single-stranded DNA is much more sensitive to damaging agents, including ROS, than double-stranded DNA, the local single-stranded nature and the enzymatic specificities of Ntg1 explain the locus-specific oxidation of bases and the double-stranded breakage at *ori*5 [62] (Figure 4). Thus, ROS act as signal mediators to regulate mtDNA replication and copy number [62].

The activity of the TCA cycle (tricarboxylic acid cycle, citric acid cycle) is regulated at the transcriptional level by nutritional and other environmental conditions [63]. The TCA cycle itself is a source of ROS production at the stage of α-ketoglutarate dehydrogenase, especially when the NADH/NAD^+^ ratio is increased [64]. Although the role of the mtDNA copy number in the regulation of mitochondrial gene expression has not been clarified, this, and the function of ROS as a mediator of mtDNA copy number control, suggest an interesting possibility: *i.e.*, the activation of the TCA cycle to produce NADH, enhances ROS production and triggers an increase in the mtDNA copy number, which in turn might increase the expression of genes encoded in the mitochondrial genome.

### 3.5. Does ρ^+^ mtDNA, Like ρ^−^ mtDNA, Replicate by the Rolling-Circle Mode in Yeast?

It is well established that yeast ρ^+^ mtDNA replicates by a Mhr1-mediated recombination-dependent mechanism, and that ρ^−^ mtDNA replicates by a rolling circle mode in *S. cerevisiae*, as described. Concatemers are the predominant form of ρ^+^ mtDNA in *Saccharomyces* yeast [65–67]. However, whether ρ^+^ mtDNA replicates by the rolling circle mode or not in yeasts is under debate due to a lack of direct evidence. Historically, mtDNA replication is initiated by RNA synthesis by a transcriptional RNA polymerase (Rpo41 in *S. cerevisiae*). Although the maintenance of ρ^+^ mtDNA requires Rpo41, Rpo41 is not required for mtDNA replication origin (including *ori5*) functions, as described above [58].

When concatemers are formed through multiple rounds of crossing-over from circular or linear monomeric DNA, DNA networks are formed during the process. *S. cerevisiae* has branched mtDNA molecules [67]. Holliday junctions are obligatory intermediates of crossing-over, and a Holliday junction resolvase is required to finish concatemer formation. A typical example is *E. coli* phage T4 DNA replication, in which branched molecules or networks of DNA are formed from permutated linear DNA molecules, and Endo VII, which resolves the Holliday junctions, is required before packaging by terminase [31]. On the other hand, *S. cerevisiae* ρ^+^ mtDNA maintenance does not depend on Cce1, the sole yeast mitochondrial Holliday junction resolvase [51,52,68], and thus, the mtDNA network detected in yeast mitochondria is not an essential intermediate of ρ^+^ mtDNA replication and partitioning in *S. cerevisiae*. Since crossing-over and rolling circle replication are the alternative pathways to form concatemers from monomeric mtDNA (the major form of mtDNA in daughter cells), this supports rolling circle replication as the major pathway of ρ^+^ mtDNA inheritance. The concatemer formation by Cce1-dependent crossing-over appears to act as a minor pathway for the maintenance of ρ^−^ mtDNA, because *mhr1-1* cells maintain a very small amount of ρ^−^ mtDNA at the restrictive temperature, and the presence of the *cce1* null mutation in the cell is required for the complete removal of mtDNA [12].

The copy number of ρ^+^ mtDNA is increased in isolated yeast mitochondria by a treatment with limited amounts of hydrogen peroxide, and this increase depends on both Mhr1 and Ntg1, as in the case of hypersuppresive ρ^−^ mtDNA [62]. The copy number of ρ^+^ mtDNA in yeast cells increased when the cells were cultured under conditions that increased intracellular ROS [62]. These results also support the proposal that ρ^+^ mtDNA replicates by the recombination-dependent mechanism, including Mhr1 and Ntg1. In addition, in the ROS-induced increase in mtDNA in ρ^+^ cells, the copy numbers of genetic markers on mtDNA distant from *ori5* or other active replication origins changed simultaneously, without a detectable difference from that of *ori5* [62]. This observation supports rolling circle replication and is inconsistent with DNA replication initiated at each mtDNA genomic unit (replicon), since, in the latter mode, the copy number of *ori5* should increase before those of the genetic markers distant from a replication origin, just after the induction of mtDNA replication by ROS.

## 4. Two Classes of Homologous Pairing Proteins, and the Choice between Rolling Circle or Double-Stranded Break-Induced DNA Replication and Simple Gene Conversion

In recombination and rolling-circle DNA replication in yeast (*S. cerevisiae*) mitochondria, D-loop formation is catalyzed by an ATP-independent homologous pairing protein, Mhr1 [12], but does not depend on RecA-family proteins. This is also the case in *Escherichia coli* bacteriophage λ and its relative, which use ATP-independent pairing proteins, Redβ and RecT, for homologous pairing, respectively [69,70]. In addition, some recombination mediators, including Rad52 [71,72], a BRCA2 homologue [73] and others [74], were found to have an ATP-independent homologous pairing activity. These ATP-independent homologous pairing proteins are structurally and evolutionally unrelated to each other (except for Redβ and RecT) and to the RecA-family proteins.

There was some debate as to whether the RecA-family proteins and the ATP-independent proteins catalyzed homologous pairing through the same or different molecular mechanisms. In all cases, the first intermediate for homologous pairing is the single-stranded DNA-protein complex. NMR analyses of the single-stranded DNA in the complex revealed that the same, uniquely extended structures were induced in the single-stranded DNA upon binding to a homologous pairing protein, independent of its origin and requirement for ATP. This means that the molecular mechanism of homologous pairing is common at the DNA level, and is independent of the requirement for ATP and the origin of the homologous pairing proteins [75].

How are the pathways subsequent to D-loop formation by homologous pairing proteins chosen, between prolonged DNA synthesis for rolling-circle replication or double-stranded break-induced DNA replication and short repair DNA synthesis, to lead to simple gene conversion and/or crossing-over? To address this question, it is important to consider that the nuclear ATP-independent homologous pairing protein, Rad52, and yeast nuclear double-stranded break-induced DNA replication are better understood than mitochondrial rolling circle replication and gene conversion. As described, unlike *RAD51* yeast cells, in which induced double-stranded breaks are generally repaired by simple gene conversion, when double-stranded breaks were induced in diploid *rad51*Δ*/rad51*Δ cells, one-third of the breaks were repaired by Rad52-dependent double-stranded break-induced DNA replication, while none were repaired by simple gene conversion [34]. It was assumed that Rad51-dependent heteroduplex joint formation at both sides of a double-stranded break forms barriers to DNA replication [76]. However, the formation of a replication barrier can be more simply explained by the nature of the RecA-family protein, without assuming heteroduplex formation on both sides of a break. The RecA spiral filament formed on a single-stranded tail extends onto double-stranded DNA from the site of homologous pairing, leaving the 3′ terminus of the paired single-stranded tail within the spiral filament [77]. In contrast, crystallographic and biochemical studies suggested that human Rad52 catalyzes homologous pairing within a groove that runs around the undecameric ring of the conserved Rad52 *N*-terminal domain [78,79]. The structure of the N-terminal domain of Rad52 is well conserved between human and yeast. The structural differences between RecA/Rad51 and Rad52 might influence the accessibility of the DNA-replication apparatus to the 3′ termini of the paired single-stranded tails, and explain why Rad52 promotes double-stranded break-induced DNA replication, while Rad51 promotes simple gene conversion and crossing over.

## 5. Evolutional Conservation of Mitochondrially-Encoded Protein Composition

Mitochondrial DNA suffers from spontaneous mutagenesis at an order of magnitude higher rate than nuclear DNA [80,81]. Hence, it was often said that “mitochondria evolve quickly” [80]. The sizes of the mtDNAs are quite different between human and *Saccharomyces* yeast (*ca.* 16 kbp in human [82] and 75-85 kbp in *S. cerevisiae* [83]). The yeast mtDNA encodes additional genes, but these are mostly non-essential for respiration functions, such as selfish genes, introns and intron-encoded proteins required for mRNA maturation and homing. A comparison of the compositions and amino acid-sequences of the proteins encoded on the mtDNA between *S. cerevisiae* and human reveals relative evolutional conservation, rather than differences (Table 1) [84], except that the yeast lacks NADH-ubiquinone oxidoreductase (Complex I). Complex I is replaced by cytosolic NADH dehydrogenase in the yeast, while other fungi, including *Neurospora crassa*, and prokaryotes have Complex I [85]. Although the respiratory chain and ATP synthase complexes consist of a number of subunits and proteins, six of them are encoded by the mtDNA, and the rest are encoded by the nuclear genome in both human and yeast. The exceptions are only two genes, *atp9* and *var1*, encoding a subunit of ATP synthase and a component of the 38S mitochondrial ribosomal subunit, respectively. In addition, the amino acid sequences of the proteins encoded on mtDNA are significantly conserved between yeast and human (Table 1).

**Table 1.** Proteins encoded by the human and the yeast mitochondrial genomes.

**Protein**	**Human Genes** **(13 Genes)**	**Yeast** ***S. cerevisiae*** ** Genes**	**% Identities,** **[% Conservative Substitutions]**
NADH-ubiquinone oxidoreductase chain 6	*MTND6 or ND6*	-	-
NADH-ubiquinone oxidoreductase chain 5	*MTND5 or ND5*	-	-
NADH-ubiquinone oxidoreductase chain 4L	*MTND4L or ND4L*	-	-
NADH-ubiquinone oxidoreductase chain 4	*MTND4 or ND4*	-	-
NADH-ubiquinone oxidoreductase chain 3	*MTND3 or ND3*	-	-
NADH-ubiquinone oxidoreductase chain 2	*MTND2 or ND2*	-	-
NADH-ubiquinone oxidoreductase chain 1	*MTND1 or ND1*	-	-
Cytochrome c oxidase polypeptide III	*MTCO3* *or COIII*	*cox 3* *or oxi2*	44% [63%]
Cytochrome c oxidase polypeptide II	*MTCO2* *or COII*	*cox 2* *or oxi1*	44% [65%]
Cytochrome c oxidase polypeptide I	*MTCO1* *or COI*	*cox1* *or oxi3*	59% [77%]
ATPase subunit 9		*atp9* *or oli1*	-
ATP synthase protein 8 (ATPase subunit 8)	*MTATP8* *or ATP8*	*atp8*	-
ATP synthase A chain (Protein 6)	*MTATP6* *or ATP6*	*atp6* *or oli2*	35% [55%]
Cytochrome *b*	*MTCYB or COB* *or CYTB*	*cob* *or cob-box*	50% [70%]
mitoribosomal polypeptide			*var1*	-
ORFs with unknown functions, intron-encoded endo DNases & maturases		*ORF1, ORF5-ORF12, I-SceII etc*	Selfish genes

This evolutional conservation in the mitochondrial genome has been generally explained by the mitochondrial-nuclear interaction as the unit of selection [86]. It would be worth considering another, but not alternative, possibility that homoplasmy contributes to the conservation of the mitochondrial genome, since homoplasmy completely neutralizes homologous recombination. Mitochondria have a number of copies of mtDNA, which quickly acquire mutations. Without this neutralization system, in cooperation with spontaneous mutagenesis, homologous gene conversion would cause the rapid diversification of the genome sequence and the acquisition of new genes in mitochondria, as observed in the DT40 cells used in the ADLib system.

## 6. Sequence Homogenization in Mammals and Human

### 6.1. Homoplasmy in Human Mitochondria

The heteroplasmy of even neutral mitochondrial genetic markers segregates within a few generations into homoplasmy in mammals [87–89]. In the traditional theory of mammalian or human mtDNA replication, at the heavy chain replication origin, RNA is synthesized by the mitochondrial RNA polymerase for transcription, processed by an endonuclease, and then heavy chain synthesis is initiated at the 3′ end of the RNA, as a primer. The heavy chain synthesis continues to the light chain replication origin associated with D-loop formation (a different D-loop than that formed by homologous pairing in recombination), and then pauses. The light chain is synthesized along the D-loop, and then the entire mtDNA is replicated [90–92]. This traditional theory was challenged by the coupled leading- and lagging-strand synthesis mechanism [93–96]. Both theories explain how a circular mtDNA monomer replicates into two circular sister monomers. However, if each mtDNA copy is duplicated independently, followed by random 34 upon cell division, then the segregation of heteroplasmic parental calls, containing hundreds to thousands of mtDNA copies, into homoplasmic progeny is difficult to explain. Therefore, extensive reduction of the mtDNA copy number may occur at a critical step for the segregation (genetic bottleneck). The existence of this genetic bottleneck is under debate, and no gene function at the bottleneck step was found in mammals [4–7].

It was once believed that mitochondrial DNA (mtDNA) does not recombine in mammals, but recently various results supporting homologous mtDNA recombination in human and mammals have been published [97–99]. Although it was only shown in ρ^−^ yeast, rolling circle replication followed by selective transmission of the replication products, concatemers, to the progeny is the sole mechanistic model with genetic and biochemical support.

### 6.2. Sequence Homogenization within Each Repeated DNA Sequence in Nuclear Genomes

In yeast mitochondria, the mtDNA can be regarded as tandem DNA repeats (*i.e.*, concatemers) of mitochondrial genome units. The nuclear genome also has various tandem DNA repeats (repeated DNA sequences or repetitive elements), including rDNAs, LINEs, SINEs and satellite DNAs. As in mitochondrial DNA, it has been recognized that each of these repeated DNA sequences consists of a homogeneous sequence, and the homogenization of the sequences has been discussed, in terms of concerted evolution [100]. Unequal crossing-over and gene conversion have been considered as mechanisms for concerted evolution. A recent study of hybrid scallops revealed that the very rapid homogenization of rDNA sequences into the maternal genotype is likely to occur by biased mitotic gene conversion [101]. Eukaryotic cells generally contain extrachromosomal circular DNA with heterogeneous sizes, consisting of sequences of repeated chromosomal DNA sequences. The findings in human cells of circular multimers of rDNA and of structures corresponding to intermediates of rolling circle DNA replication suggest a mechanism for the concerted evolution or homogenization of tandem repeats [102]. Telomeres consist of repeated sequences, and in the absence of telomerase, the telomere is maintained by Rad52-dependent and Rad51-independent rolling circle replication in yeast [36]. Therefore, it would be interesting to consider the possibility that cells use rolling circle DNA replication as a mechanism to homogenize (or homoplasmy) repeated DNA sequences, in both the nuclei and mitochondria of various eukaryotes.

## 7. Conclusion

As observed in the immunoglobulin loci in DT40 cells, gene conversion among a DNA sequence family consisting of similar, but varying, sequences extensively diversifies the sequences and even efficiently generates genes for new functional proteins. Each cell has hundreds to thousands of copies of mtDNA, and thus mtDNA can be regarded as a DNA sequence family. MtDNA acquires mutations ten-times more rapidly than nuclear DNA, and is subjected to homologous gene conversion (gene conversion is observed in yeast mitochondria, and evidence is accumulating for recombination in mammalian mtDNA). Thus, it could be speculated that mitochondria have a heterogeneous mtDNA population and that the mitochondrial genome evolved much more quickly than the nuclear genome. However, the heterogeneous mtDNA population (heteroplasmy) segregates within several cell division cycles in yeast, or a few sexual generations in mammals, into a single genotype within a cell, in a phenomenon called homoplasmy, and the mitochondrial genome is significantly conserved between yeast and human.

The following results were obtained in yeast *Saccharomyces cerevisiae* ρ^−^ cells: (1) The major form of mtDNA is concatemers, but growing buds predominantly have monomeric circular mtDNA; (2) rolling circle DNA replication is the major pathway to produce concatemers, which are selectively transmitted to daughter cells; (3) as in gene conversion and crossing-over, rolling circle mtDNA replication is initiated by a double-stranded break and follows homologous pairing (D-loop formation) by Mhr1; (4) a double-stranded break to initiate rolling circle replication is introduced at a replication origin, *ori5*, by Ntg1; and (5) the mtDNA copy number is very likely to be controlled by Ntg1-mediated double stranded breakage, according to the amounts of ROS (reactive oxygen species), which act as signaling mediators.

These mechanisms readily explain the observed quick segregation of heteroplasmic ρ^+^ cells to generate homoplasmic ρ^+^ cells within several cell-division cycles and the responses of ρ^+^ mitochondria to oxidative stress. Biochemical and genetic data support the functions of these mechanisms in ρ^+^ yeast cells. Critical support for this mechanism in mammals awaits the finding of a mammalian homologue of Mhr1.

A possible factor for the selection between simple gene conversion (with or without crossing-over) and recombination-dependent DNA replication (rolling circle replication and double-stranded break induced DNA replication), as a consequence of double-stranded cleavage and the subsequent homologous pairing (D-loop formation), is the 3D structures of the proteins that catalyze D-loop formation. RecA-family proteins prefer gene conversion, and ATP-independent homologous pairing proteins select recombination-dependent DNA replication along with gene conversion. These classes of proteins differ in terms of their modes of polymerization along DNA substrates and products.

Finally, rolling circle DNA replication may be a general mechanism for sequence homogenization (homoplasmy) of multiple DNA copy systems and tandem DNA repeats, in both the mitochondria and nuclei of various eukaryotic cells.
